# *NDUFB6* Polymorphism Is Associated With Physical Activity-Mediated Metabolic Changes in Type 2 Diabetes

**DOI:** 10.3389/fendo.2021.693683

**Published:** 2021-09-24

**Authors:** Dominik Pesta, Tomas Jelenik, Oana-Patricia Zaharia, Pavel Bobrov, Sven Görgens, Kálmán Bódis, Yanislava Karusheva, Nina Krako Jakovljevic, Nebojsa M. Lalic, Daniel F. Markgraf, Volker Burkart, Karsten Müssig, Birgit Knebel, Jörg Kotzka, Jürgen Eckel, Klaus Strassburger, Julia Szendroedi, Michael Roden

**Affiliations:** ^1^ Institute for Clinical Diabetology, German Diabetes Center, Leibniz Center for Diabetes Research at Heinrich Heine University, Düsseldorf, Germany; ^2^ German Center for Diabetes Research (DZD e.V.), Düsseldorf, Germany; ^3^ Institute of Aerospace Medicine, German Aerospace Center (DLR), Cologne, Germany; ^4^ Center for Endocrinology, Diabetes and Preventive Medicine (CEDP), University Hospital Cologne, Cologne, Germany; ^5^ Cologne Excellence Cluster on Cellular Stress Responses in Aging-Associated Diseases (CECAD), Cologne, Germany; ^6^ Institute for Biometrics and Epidemiology, German Diabetes Center, Leibniz Center for Diabetes Research at Heinrich-Heine University, Düsseldorf, Germany; ^7^ Paul-Langerhaus-Group Integrative Physiology, German Diabetes Center, Düsseldorf, Germany; ^8^ Department of Endocrinology and Diabetology, Medical Faculty and University Hospital, Heinrich Heine University Düsseldorf, Düsseldorf, Germany; ^9^ Clinics for Endocrinology, Diabetes and Metabolic Diseases, University Clinical Centre of Serbia, Faculty of Medicine, University of Belgrade, Belgrade, Serbia; ^10^ Institute of Clinical Biochemistry and Pathobiochemistry, German Diabetes Center, Leibniz Center for Diabetes Research at Heinrich-Heine University, Düsseldorf, Germany

**Keywords:** diabetes mellitus, physical activity, single nucleotide polymorphism, insulin sensitivity, mitochondrial function

## Abstract

The rs540467 SNP in the *NDUFB6* gene, encoding a mitochondrial complex I subunit, has been shown to modulate adaptations to exercise training. Interaction effects with diabetes mellitus remain unclear. We assessed associations of habitual physical activity (PA) levels with metabolic variables and examined a possible modifying effect of the rs540467 SNP. Volunteers with type 2 (n=242), type 1 diabetes (n=250) or normal glucose tolerance (control; n=139) were studied at diagnosis and subgroups with type 1 (n=96) and type 2 diabetes (n=95) after 5 years. Insulin sensitivity was measured by hyperinsulinemic-euglycemic clamps, oxygen uptake at the ventilator threshold (VO_2_AT) by spiroergometry and PA by questionnaires. Translational studies investigated insulin signaling and mitochondrial function in *Ndufb6* siRNA-treated C2C12 myotubes, with electronic pulse stimulation (EPS) to simulate exercising. PA levels were 10 and 6%, VO_2_AT was 31% and 8% lower in type 2 and type 1 diabetes compared to control. Within 5 years, 36% of people with type 2 diabetes did not improve their insulin sensitivity despite increasing PA levels. The *NDUFB6* rs540467 SNP modifies PA-mediated changes in insulin sensitivity, body composition and liver fat estimates in type 2 diabetes. Silencing *Ndufb6* in myotubes reduced mitochondrial respiration and prevented rescue from palmitate-induced insulin resistance after EPS. A substantial proportion of humans with type 2 diabetes fails to respond to rising PA with increasing insulin sensitivity. This may at least partly relate to a polymorphism of the *NDUFB6* gene, which may contribute to modulating mitochondrial function.

**Clinical Trial Registration:**

ClinicalTrials.gov, identifier NCT01055093. The trial was retrospectively registered on 25^th^ of January 2010.

## Introduction

Exercise training improves insulin sensitivity and ameliorates the risk of diabetes onset and cardiovascular mortality in type 2 diabetes (T2D) (1–3), but may also be beneficial for type 1 diabetes (T1D) (4). Nevertheless, the effects of structured exercise training, i.e. aerobic or resistance training, vary considerably (5), with up to 63% of participants being non-responders with regard to improvements of glucose homeostasis or cardiovascular outcomes (5, 6). This phenomenon and its causes have not yet been addressed in a prospective manner in newly-diagnosed diabetes.

Impaired response to exercise training may not only result from acquired, but also from inherited factors (7, 8). We previously demonstrated that an SNP in the NADH dehydrogenase-1ß subcomplex subunit 6 (*NDUFB6*) of the mitochondrial complex I relates to impaired muscle mitochondrial plasticity after exercise training in first-degree relatives of type 2 diabetic patients (7, 9). Presence of the G/G genotype (rs540467) was associated with exercise-mediated increases in muscle ATP synthase flux (7) and insulin sensitivity (9) with responders to exercise being more frequently carriers of the G allele of the NDUFB6 rs540467 polymorphism. These associations remain unclear in patients with diabetes mellitus.

Increasing habitual physical activity (PA), i. e. any type of muscular activity leading to increased energy expenditure, lowers the risk of diabetes in adults with impaired fasting glucose (10) and associates with reductions in liver fat content (11). PA could therefore represent an attractive alternative to structured exercise training for diabetes patients for improving insulin sensitivity and body composition. Despite growing evidence for non-responsiveness to exercise interventions, less is known about a possible non-response to habitual PA in patients with diabetes mellitus. Particularly in these patients, it is unclear whether (i) lower habitual PA effects metabolism, e. g. insulin sensitivity, body composition and lipid metabolism at time of diagnosis and during disease progression, (ii) the responder/non-responder status also extends to habitual PA-mediated beneficial effects and if so, (iii) whether responsiveness of PA-mediated improvements in metabolism relate to a gene polymorphism and to gene-related metabolic effects in T2D.

Using comprehensive phenotyping and based on previous observations, we performed a longitudinal analysis of how the polymorphism influences the relationship between changes in PA and changes in metabolism over five years in people with diabetes mellitus and healthy humans and the underlying cellular mechanisms in a muscle cell culture model suitable to assess effects of PA *in vitro* (12).

## Materials and Methods

The prospective observational German Diabetes Study (GDS) monitors people with recent-onset diabetes and glucose-tolerant humans (ClinicalTrial.gov registration no: NCT01055093) (13). Volunteers consented to a protocol, approved by the ethics board of Heinrich-Heine-University Düsseldorf and performed according to the 2013 version of the Declaration of Helsinki. Inclusion and exclusion criteria of the GDS applied (13). This longitudinal analysis included participants enrolled into GDS between 02/2009 and 04/2020 with complete data sets regarding the relevant variables.

### Participants

Individuals with recently diagnosed type 1 (n=250), type 2 (n=242) or without diabetes (control, n=139) were included. From these collectives, a subgroup of individuals with type 1 (n=96) and type 2 diabetes (n=95) was available for the 5-years follow-up analysis. As the GDS is an ongoing observational study, by design, 48% of patients have not reached the follow-up time point of 5 years disease duration at the time of analysis. The “true” loss to follow-up is ~13% (14) and lies within the range of comparable cohorts (15, 16). All metabolic tests were performed under the following conditions: (i) carbohydrate-rich nutrition for 3 days, (ii) a physical activity break of 48 hours and (iii) overnight fasting for 10-12 hours before the test.

### Physical Activity and Dietary Intake Reporting

Self-reported PA levels over the preceding 12 months were assessed by the German version (17) of the modified Baecke questionnaire, validated for people with diabetes (18) which showed good agreement with triaxial-accelerometry and are valid for larger cohort studies (19). The Baecke questionnaire inquiries about sport and leisure type of activity as well as activity at work on a 5-point scale. The higher the score, the higher the level of PA. The combined Baecke score was calculated as the sum of the sports-, leisure- and work score. The EPIC-Potsdam Food Frequency Questionnaire was used to assess dietary intake in a subgroup with type 2 diabetes (n=37) (20). This questionnaire assesses average food intake during the past 12 months and allows for estimating macronutrient and total energy intake. After diabetes diagnosis, physicians give general lifestyle recommendations to their patients to improve their physical activity levels, but there was no structured lifestyle intervention provided.

### Spiroergometry

All participants performed an incremental exhaustive exercise test on a cycle ergometer (Viasprint 200, Ergoline, Bitz, Germany) for recording VO_2_peak and VO_2_ at the aerobic threshold (VO_2_AT). VO_2_AT was assessed using the V-slope method (21). As an index of physical fitness, VO_2_AT has repeatedly been shown to better predict maximal endurance performance (22). Exhaustion was defined according to the guidelines on cardiopulmonary exercise testing by the presence of one or more of the following criteria: (i) respiratory exchange ratio (RER) >1.15 or if (ii) predicted maximum heart rate (HRmax), (iii) predicted VO_2_peak and/or a plateau, or (iv) predicted maximal work rate were achieved (23). Participants not achieving the exhaustion criteria were excluded from the analysis.

### Bioimpedance Analysis (BIA)

BIA was carried out after an overnight fast and was used for the estimation of fat mass (FM), percent fat mass (%FM) and fat-free mass (BioElectrical Impedance Analyzer System, RJL Systems, Detroit, MI).

### Modified Botnia Clamp Test

This metabolic test was used to assess insulin sensitivity and secretion after overnight fasting and a physical activity break of 48 hours (13). An i. v. glucose tolerance test was performed for 60 minutes to measure total C-peptide secretion from the incremental area under the curve for C-peptide levels. Then a hyperinsulinemic-eugycemic clamp using isotopic dilution ([6,6-^2^H_2_]glucose) was performed to assess whole-body insulin sensitivity. The hyperinsulinemic-eugycemic clamp test was started with a priming insulin dose (10 mU per kg body weight per min for 10 minutes i.v.; Insuman Rapid, Sanofi, Frankfurt, Germany) followed by a constant insulin infusion (1.5 mU per kg body weight per min) over a 3-hours period. Blood glucose concentration was maintained at 90 mg/dl by a variable i. v. infusion of 20% glucose. Glucose infusion rates during steady state of the clamp were used to calculate whole-body insulin sensitivity and expressed as M value after glucose space correction (13).

### Glucagon Stimulation Test

For this metabolic test, volunteers received an i. v. bolus of 1 mg glucagon to obtain a measure of glucagon-stimulated C-peptide (insulin) secretion capacity from difference of C-peptide (insulin) concentrations at 6 and 0 minutes (13).

### Magnetic Resonance (MR) Spectroscopy

A subgroup of people with type 2 diabetes (n=18) underwent ^1^H MR spectroscopy using a stimulated echo acquisition mode (STEAM) sequence in a 3-T MR scanner (Achieva X-series, Philips Healthcare, Best, Netherlands), to assess hepatocellular lipid content (24).

### Fatty Liver Index (FLI) and Homeostatic Model Assessment for Insulin Resistance (HOMA-IR)

The FLI was calculated from BMI, waist circumference, serum triglycerides and gamma-glutamyl transferase as a noninvasive surrogate index of hepatic steatosis (25). HOMA-IR was calculated according to the formula: fasting insulin (µU/l) * fasting glucose (mmol/1)/22.5 (26).

### Genotyping

Genomic DNA was extracted from whole blood (27) and genotyping was conducted using real-time polymerase chain reaction-based allelic discrimination according to manufacturer’s recommendations with probe-based genotyping assays for the single-nucleotide polymorphism rs540467 in the *NDUFB6* gene (Life Technologies, Darmstadt, Germany). The genotype concordance of >99.8% was determined using TaqMan Genotyper software v.1.3 (Life Technologies). All variants were in Hardy-Weinberg-equilibrium and re-sequencing of 1% of randomly chosen individuals was conducted for data validation and quality management, confirming genotyping results by 100%. Due to the low number of AA carriers, A allele carriers (GA and AA) were combined for analyses.

### Cell Culture and siRNA Transfection

C2C12 myoblasts (American Type Culture Collection, Manassas, VA, USA) were grown in DMEM (Gibco, Berlin, Germany), 10% FBS and antibiotics (100 U/mL penicillin and 100 mg/mL streptomycin) in humid air (5% CO_2_, 37°C) and differentiated into myotubes by switching to DMEM with 2% horse serum (Gibco, Berlin, Germany) up to 6 days (28). Silencing experiments in differentiated C2C12 myotubes were performed using FlexiTube small interfering RNA (siRNA) and HiPerfect (Qiagen) according to the manufacturer’s instructions. Control cells were treated with negative control siRNA (AllStars Negative Control siRNA, Qiagen, Hilden, Germany). Cells were studied under basal or palmitate-treated (0.2 mM, 24 h) conditions, with and without electronic pulse stimulation (EPS) contraction (n=4-6) (29). EPS (1 Hz, 2 ms and 11.5 V) was applied for 24 h to fully differentiated C2C12 myotubes (day 6) in six-well dishes using a C-Dish combined with a pulse generator emitting bipolar stimuli (C-Pace 100; IonOptix, Milton, MA, USA) (29). Cells that were not stimulated, but were incubated with a C-Dish for the same timepoint (24 h), were used as controls. Insulin signaling was assessed after 10 min stimulation with 100 nM insulin (I5523, Sigma, Munich, Germany).

### High-Resolution Respirometry

Mitochondrial function was assessed by high-resolution respirometry in 0.25*10^6^/ml digitonin-permeabilized myotubes (Oxygraph-2k, Oroboros Instruments, Innsbruck, Austria) (30). Complex I-linked leak respiration was assessed using malate (2 mM), pyruvate (10 mM) and glutamate (10 mM) and β-oxidation-linked electron-transferring flavoprotein complex (CETF)-respiration using malate (2 mM) and octanoyl-carnitine (0.2 mM). State 3 respiration was induced in both protocols by addition of ADP (2.5 mM), state 4o by oligomycin (5 µM), maximal respiration (state u) by stepwise increments of 0.25 µM carbonyl-cyanide-p-trifluoromethoxyphenylhydrazone (FCCP) (30). Respiratory control ratio (RCR) was calculated as state 3 over state 4o, leak control ratio (LCR) as leak state over state 4o.

### Quantitative Real-Time (qRT) PCR

Total RNA was isolated using RNeasy kit (Qiagen, Hilden, Germany). mRNA expression was measured using pre-designed primers (Quantitect Primer Assay, Qiagen) and GoTaq qPCR Master Mix (Promega, Mannheim, Germany) in SYBR Green-based qRT PCR.

### Western Blots

Reagents for SDS-PAGE were supplied by GE Healthcare (Munich, Germany) and Sigma and rotiphorese by Carl Roth (Karlsruhe, Germany). Antibodies used were anti-phosphorylated Akt at serine 473 (pAkt-Ser473; Cell Signalling Technology), ß-actin (Sigma) and horseradish peroxidase-conjugated goat anti-rabbit and anti-mouse IgG (Promega, Mannheim, Germany).

### Laboratory Analyses

For all parameters [HbA1c, plasma glucose, C-peptide, lipids, high-sensitivity C-reactive protein (hsCRP)], fasting blood samples were taken at baseline and after 5 years using standardized tubes and tube additives and analyzed using identical instruments and methods as described previously (13).

### Statistics

Data are presented as percentages (%), mean ± SD or median [25th, 75th percentiles] in case of skewed distributed variables, as appropriate. For group comparisons, analyses were adjusted for age, sex and BMI. The Tukey-Kramer method was used to adjust p-values for multiple comparisons or pairwise comparisons of three groups.

Univariate and multivariable linear least squared regression was performed to analyze the association of PA levels and other variables. In multivariable analyses, regression coefficients were adjusted for age, sex and BMI. To investigate whether the slope between NDUF genotypes differ, an interaction term of the genotype (dominant coding) and the independent variable were added to the model and tests for interaction were performed. Given a sample size of n=90, small to medium effect sizes (Cohens f=0.08) can be detected with a power of 80% (31). Fisher’s exact test was used to compare categorical variables. For skewed distributed variables (M-value, fasting C-peptide, fasting triglycerides, hsCRP), data was log-transformed before analyses. P-values ≤0.05 after correction for multiple testing were considered to indicate significant differences. Statistical analyses were performed with SAS (version 9.3; SAS Institute, Cary, NC) and figures were computed with Graph Prism (version 7.04 for Windows; GraphPad Software, La Jolla California USA).

## Results

### Physical Activity, Exercise Capacity and Metabolism in Recent-Onset Diabetes (Baseline)

According to diabetes type, age and BMI differed between groups (**Table 1**) so that all further statistical analyses were adjusted for these confounders. Waist-to-hip ratio (WHR) and %FM were highest in T2D compared to T1D and glucose-tolerant persons. The combined Baecke index was 6% and 10% lower in T1D and T2D compared to glucose-tolerant individuals (controls). Similarly, the sports index was also lower in the diabetes groups, while leisure and work indices were not different between groups. VO_2_AT was 14% and 23% lower in T1D and T2D compared to controls, respectively.

**Table 1:** Baseline characteristics of healthy controls (n=84), patients with type 1 (n=212) and type 2 (n=215) diabetes.

Parameter	Control	Type 1 Diabetes	Type 2 Diabetes
Age (years)	43 ± 14	35 ± 11^†^	51 ± 10^†§^
Sex (% female)	26	40	33
rs540467 (GG/GA/AA %)	52/42/6	61/32/7	60/35/5
Body weight (kg)	86.6 ± 18.5	76.6 ± 14.3^†^	92.2 ± 19.0^*§^
BMI (kg.m^-2^)	27.3 ± 4.9	24.8 ± 3.9^†^	30.3 ± 5.3^†§^
Fat mass (%)	27.2 ± 7.9	24.4 ± 8.2^†^	33.1 ± 7.6^†§^
Waist-to-hip ratio	0.90 ± 0.08	0.87 ± 0.09^†^	0.96 ± 0.07^†§^
Baecke Index	8.9 ± 1.4	8.4 ± 1.4^†^	8.0 ± 1.4^†^
Sports Index	3.2 ± 0.8	3.0 ± 0.9^*^	2.7 ± 0.8^†^
Leisure Index	3.2 ± 0.7	3.1 ± 0.7	3.0 ± 0.7
Work Index	2.4 ± 0.6	2.3 ± 0.6	2.4 ± 0.7
VO_2_AT (ml.min^-1^.kg^-1^)	19.1 ± 5.3	17.6 ± 5.9^†^	13.2 ± 3.7^†§^
M-value (mg.kg^-1^.min^-1^)	11.0 [8.5;12.7]	8.4 [6.6;10.4]^†^	6.5 [4.7;8.4]^†‡^
Fasting blood glucose (mg.dl^-1^)	89.5 ± 14.9	131.5 ± 37.7^†^	128.6 ± 46.0^†^
HbA1c [%; (mmol.mol^-1^)]	5.2 ± 0.3; [33 ± 3.3]	6.5 ± 1.0; [48 ± 10.9]^†^	6.4 ± 0.8; [46 ± 0.3]^†^
HOMA-IR	632.8 ± 422.5	2154.5 ± 3448.3^†^	2212.5 ± 1850.8^†^
Glucagon-stimulated C-peptide secretion (ng.ml^-1^)	3.9 ± 1.7	0.8 ± 1.0^†^	3.0 ± 1.5^†§^
Total C-peptide secretion (ng.ml^-1^)	183.9 [133.4;221.1]	16.6 [6.4;34.1]^†^	99.5 [58.9;148.5]^†§^
Total cholesterol (mg.dl^-1^)	196 ± 35	183 ± 36	203 ± 43
LDL-cholesterol (mg.dl^-1^)	124 ± 34	109 ± 30	132 ± 37
HDL-cholesterol (mg.dl^-1^)	60 ± 19	61 ± 17	47 ± 13^†§^
Fasting triglycerides (mg.dl^-1^)	87 [60;130]	74 [55;104]	127 [96;192]^†§^
ALT (U.l^-1^)	23 [17;30]	20 [16;26]	28 [21;41]^*‡^
FLI (a. u.)	35 ± 30	24 ± 24	66 ± 28^†§^
hsCRP (mg.dl^-1^)	0.1 [0.1;0.2]	0.1 [0.1;0.2]	0.2 [0.1;0.4]^†‡^

Data are given as percentages for categorical variables, mean ± standard deviations (SD) or median [25^th^, 75^th^ percentile] for continuous variables. Significant differences as determined by one-way ANOVA are adjusted for age, sex and BMI (except for the first 6 variables) and denoted as ^*^ vs control p<0.05; ^†^ vs control p<0.01; ^‡^ vs type 1 diabetes p<0.05; ^§^ vs type 1 diabetes p<0.01; p-values are adjusted for pairwise comparisons of three groups using a step-down closed test procedure; ALT, alanine aminotransferase; FLI, fatty liver index; HbA1c, glycated hemoglobin A1c; HOMA-IR, Homeostatic Model Assessment for Insulin Resistance; hsCRP, high-sensitivity C-reactive protein.

Whole-body insulin sensitivity (M-value) was 20% and 40% lower in T1D and T2D compared to controls, respectively. The same held true for HOMA-IR, a surrogate parameter of insulin resistance. Beta-cell function (glucagon- and glucose-stimulated C-peptide secretion) was lower in T1D compared to the other groups. Glycemic control (fasting blood glucose, HbA1c) was similarly excellent in both diabetes groups, but - as expected - higher than in controls. Compared to both T1D and controls, persons with T2D had lower HDL-cholesterol, but higher triglycerides. Alanine aminotransferase (ALT), FLI and hsCRP levels were highest in T2D.

### Five-Year Follow-up of Physical Activity, Exercise and Metabolic Performance

**Figure 1:**
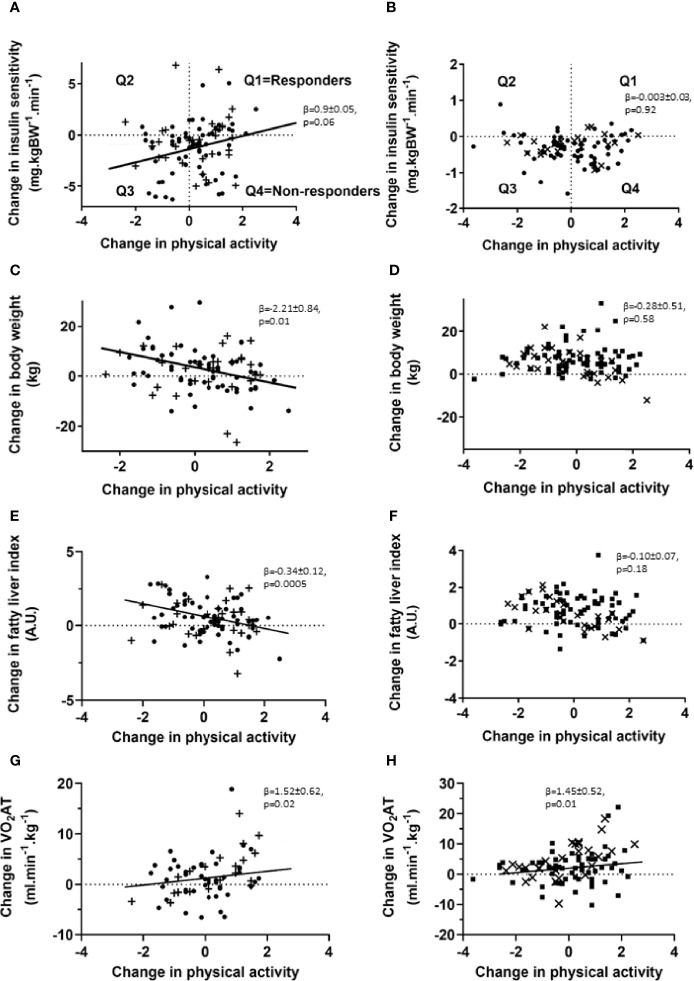
Association of changes in PA levels and insulin sensitivity, body weight, liver fat estimates and exercise capacity in individuals with type 2 and type 1 diabetes. Association of changes in PA levels and insulin sensitivity **(A, B)**, body weight **(C, D)**, liver fat estimates **(E, F)** and physical performance **(G, H)** for patients with type 2 (left panel, • – G/G allele carriers; + - G/A and A/A allele carriers) and type 1 diabetes (right panel, ▪ – G/G allele carriers; x – G/A and A/A allele carriers) at the 5-year follow-up time point. Patients with type 2 and type 1 diabetes were subdivided into four groups (Q1, Q2, Q3, Q4) based on response to changes in insulin sensitivity and PA levels. Univariate and multivariable linear least squared regression method was performed, adjusted for baseline values of both PA levels and the investigated clinical parameter. N=95 for changes in insulin sensitivity, N=95 for body weight changes and N=67 for performance changes in individuals with type 2 diabetes and N=100 for changes in insulin sensitivity, N=101 for body weight changes and N=90 for performance changes in individuals with type 1 diabetes. VO_2_ AT: oxygen uptake at the first ventilatory threshold.

Regression analyses of the changes over the 5-years follow-up revealed a trend for an association of changes in PA over 5 years and changes in M-value in TD2 (**Figure 1A**), but not T1D (**Figure 1B**). However, changes in PA levels neither affected HbA1c nor insulin secretion in individuals with T2D. Changes in PA levels, as assessed from combined Baecke and leisure indices, associated with lower body weight (BW) in type 2, but not T1D (**Figures 1C, D**). In T2D only, increased PA associated negatively with the FLI (**Figures 1E, F**), whereas increased PA was positively associated with VO_2_AT in both diabetes types (**Figures 1G, H**). In support of this observation, we found a trend of a negative association between changes in ^1^H MRS-measured hepatocellular lipid content and changes in PA in a subgroup of 18 people (p=0.06, β=-4.35 ± 2.10). Lower fasting plasma glucose associated with higher PA and leisure activity (p=0.01, β=-11.3 ± 4.4) and higher HDL-cholesterol levels related to increased PA (p=0.02, β =1.85 ± 0.75) in T2D. Furthermore, increased PA over 5 years associated with lower FM and lower waist circumference in T2D (all p<0.05). In order to assess responder status based on changes in insulin sensitivity in response to changes in PA levels, we divided individuals with type 2 and type 1 diabetes into four groups, Q1, Q2, Q3 and Q4 (**Figures 1A, B**). During the 5-year period within T2D, group 1 (Q1; n=23) increased their PA levels and insulin sensitivity, group 2 (Q2; n=8) decreased PA levels and worsened fasting blood glucose, group 3 (Q3; n=26), decreased both insulin sensitivity and PA levels and group 4 (Q4; n=37) and increased PA levels without any improvement in insulin sensitivity. Members of Q4, representing 36% of the whole cohort, were therefore defined as non-responders with regard to this endpoint.

### Acquired and Inherited Factors Determining Responder Status to Increases in Physical Activity

We then assessed changes of metabolic parameters within these groups over the 5-year period (**Table 2**). Within T2D, Q1 - in whom both PA indices and insulin sensitivity improved - decreased their fasting C-peptide, ALT and hsCRP levels. Q2 only showed increased LDL-cholesterol levels and decreased C-peptide secretion. Notably, in Q3 - where all PA indices and insulin sensitivity worsened - most anthropometric and metabolic parameters, including BW, BMI, FM, fasting plasma glucose, HbA1c, total C-peptide secretion, triglycerides and hsCRP deteriorated over the 5-year period. In Q4, fasting plasma glucose, HbA1c and total C-peptide secretion deteriorated, despite improvements of PA indices and VO_2_AT. Among T1D, Q1 increased their HbA1c and combined Baecke and sports index as well as VO_2_AT while decreasing their fasting C-peptide and total C-peptide secretion levels. Q2 improved their VO_2_AT and BW, but decreased their fasting C-peptide, glucagon-stimulated C-peptide and total C-peptide secretion levels as well as their combined Baecke and leisure index. Interestingly, in Q3, BW, BMI, diastolic blood pressure, and %FM as well as fasting plasma glucose, HbA1c, fasting C-peptide, glucagon-stimulated C-peptide secretion, total C-peptide secretion and triglycerides deteriorated over 5 years. Q4 showed an increase in BW, BMI, HbA1c, %FM and VO_2_AT and a deterioration of beta cell function (**Table 3**).

**Table 2:** Subgroup analysis of changes (Δ) of anthropometric, performance and metabolic parameters based on response to changes in whole-body insulin sensitivity and PA participation over 5 years for patients with type 2 diabetes, divided into responders (n=17), Q2 (n=5), Q3 (n=23) and non-responders (n=30).

Parameter	Responders (Q1)	Q2	Q3	Non-Responders (Q4)
Δ BMI (kg.m^-2^)	-1.0 ± 2.3	-1.1 ± 2.8	1.6 ± 2.7*	0.4 ± 2.7
Δ Body weight (kg)	-3.5 ± 8.3	-3.7 ± 8.2	4.7 ± 9.2*	0.8 ± 7.6
Δ Fat mass (%)	-0.3 ± 2.5	-0.2 ± 6.2	1.5 ± 2.7*	1.2 ± 5.2
Δ Baecke index	1.1 ± 0.6**	-1.3 ± 0.79*	-0.8 ± 0.6**	0.7 ± 0.5**
Δ Sports index	0.6 ± 0.5**	-0.5 ± 0.8	-0.3 ± 0.6*	0.4 ± 0.6**
Δ Leisure index	0.4 ± 0.5*	-0.5 ± 0.7	-0.4 ± 0.3**	0.2 ± 0.6
Δ Work index	0.2 ± 0.4*	-0.2 ± 0.2	-0.1 ± 0.5	0.2 ± 0.4*
Δ VO_2_AT (ml.min^-1^.kg^-1^)	4.1 ± 6.4	1.2 ± 2.8	0.1 ± 3.6	1.7 ± 3.5*
Δ M-value (mg.kg^-1^.min^-1^)	0.2 [0.1;0.5]**	0.2 [0.1;0.2]	-0.4 [-0.7;-0.2]**	-0.3 [-0.5;0.1]**
Δ Fasting plasma glucose (mg.dl^-1^)	7.5 ± 40.9	17.0 ± 18.8	44.8 ± 34.0**	26.7 ± 46.5**
Δ HbA1c (%; [mmol.mol^-1^])	0.2 ± 0.7; [2 ± 8]	-0.3 ± 1.1; [-3 ± 12]	0.8 ± 1.0; [9 ± 11]**	0.7 ± 1.0; [8 ± 11]**
Δ Glucagon-stimulated C-peptide secretion (ng.ml^-1^)	-0.8 ± 3.1	0.2 ± 1.1	0.3 ± 1.5	0.0 ± 1.3
Δ Total C-peptide secretion (ng.ml^-1^)	-0.3 [-0.7;0.4]	-0.2 [-0.4;-0.1]	-0.5 [-0.9;-0.2]**	-0.5 [-0.7;0.1]**
Δ LDL-cholesterol (mg.dl^-1^)	7.4 ± 24.7	26.2 ± 12.8*	12.4 ± 38.8	12.9 ± 35.8
Δ HDL-cholesterol (mg.dl^-1^)	3.2 ± 6.2*	2.6 ± 9.3	-1.8 ± 8.9	2.9 ± 7.7*
Δ Fasting triglycerides (mg.dl^-1^)	0.2 [0.0;0.4]	0.3 [0.0;0.4]	0.3 [0.1;0.5]**	0.0 [-0.2;0.4]
Δ ALT (U.l^-1^)	-0.2 [-0.6;-0.1]*	-0.2 [-0.3;0.3]	0.2 [-0.1;0.5]*	0.0 [-0.3;0.2]
Δ hsCRP (mg.dl^-1^)	-0.3 [-0.9;0.1]*	-0.7 [-1.2;-0.4]	-0.2 [-0.7;0.2]*	-0.1 [-0.6;0.1]

Data are given as percentages for categorical variables, mean ± standard deviations (SD) or median [25^th^, 75^th^ percentile] for continuous variables. Significant differences as determined by paired t-test are marked as * for p<0.05 and ** for p<0.01; for variables with skewed distribution (M-value, fasting C-peptide, fasting triglycerides, hsCRP) the p-values refer to log-transformed data; ALT, alanine aminotransferase; HbA1c, glycated hemoglobin; hsCRP, high-sensitivity C-reactive protein; PA, physical activity.

**Table 3:** Subgroup analysis of changes (Δ) of anthropometric, performance and metabolic parameters based on response to changes in whole-body insulin sensitivity and PA participation over 5 years for patients with type 1 diabetes, divided into Q1 (n=9), Q2 (n=5), Q3 (n=33) and Q4 (n=34).

Parameter	Q1	Q2	Q3	Q4
Δ BMI (kg.m^-2^)	1.1 ± 1.6	1.2 ± 1.5	2.1 ± 1.5**	1.6 ± 2.4**
Δ Body weight (kg)	3.2 ± 4.3*	5.8 ± 5.1	6.8 ± 4.9**	5.9 ± 8.0**
Δ Diastolic blood pressure (mmHg)	1.6 ± 9.3	-4.5 ± 8.0	3.6 ± 7.9*	1.2 ± 7.1
Δ Fat mass (%)	1.3 ± 6.8	1.7 ± 4.5	3.7 ± 3.0**	3.0 ± 4.3**
Δ Baecke index	1.2 ± 0.6**	-1.6 ± 1.0*	-0.8 ± 0.6**	0.8 ± 0.6**
Δ Sports index	1.0 ± 0.8*	-0.7 ± 0.7	-0.3 ± 0.4**	0.2 ± 0.5*
Δ Leisure index	0.4 ± 0.5	-0.8 ± 0.2**	-0.4 ± 0.4**	0.4 ± 0.5**
Δ Work index	-0.1 ± 0.3	-0.1 ± 0.7	-0.1 ± 0.5	0.26 ± 0.5**
Δ VO_2_AT (ml.min^-1^.kg^-1^)	4.4 ± 8.9;	2.7 ± 4.5	0.7 ± 3.6	4.4 ± 5.8**
Δ Fasting plasma glucose (mg.dl^-1^)	16.4 ± 44.5	-30.0 ± 65.3	31.4. ± 61.4**	18.4 ± 70.6
Δ HbA1c (%; [mmol.mol^-1^])	0.9 ± 0.9; [10 ± 10]*	0.0 ± 0.9; [0 ± 10]	0.6 ± 1.1; [7 ± 12]*	0.2 ± 1.2; [2 ± 13]
Δ Fasting C-peptide (ng.ml^-1^)	-1.3 [-1.9;-1.0]**	-1.5 [-2.0;-0.5]	-1.3 [-2.0;-0.6]**	-1.3 [-1.7;-0.7]**
Δ Glucagon-stimulated C-peptide secretion (ng.ml^-1^)	-0.2 [-0.5;0.0]	-0.5 [-0.5;-0.2]	-0.4 [-0.6;-0.3]*	-0.5 [-0.7;-0.2]**
Δ Total C-peptide secretion (ng.ml^-1^)	-0.9 [-1.0;-0.8]*	-0.9 [-0.9;-0.8]**	-1.1 [-2.1;-0.6]**	-1.3 [-1.8;-1.0]**
Δ Fasting triglycerides (mg.dl^-1^)	0.0 [-0.3;0.3]	-0.4 [-0.5;0.0]	0.3 [0.0;0.5]**	0.2 [-0.3;0.4]

Data are given as percentages for categorical variables, mean ± standard deviations (SD) or median [25^th^, 75^th^ percentile] for continuous variables. Significant differences as determined by paired t-test are marked as * for p < 0.05 and ** for p < 0.01; for variables with skewed distribution (M-value, fasting C-peptide, fasting triglycerides, hsCRP) the p-values refer to log-transformed data; HbA1c, glycated hemoglobin; PA, physical activity.

### PA-Mediated Metabolic Effects Stratified by Genotype

**Figure 2:**
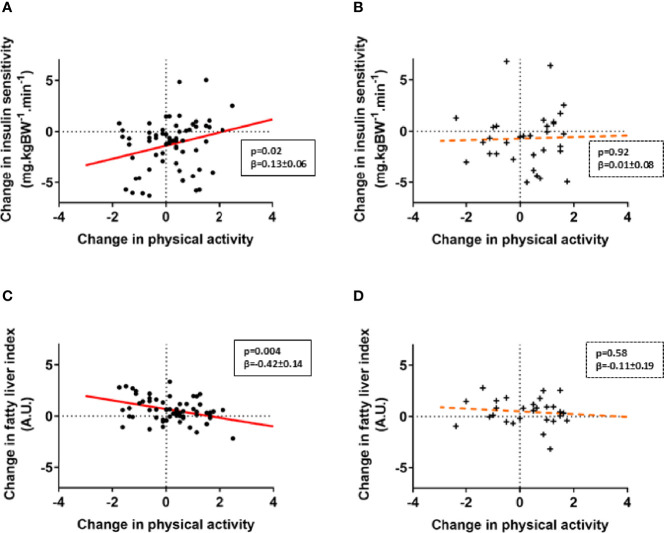
*NDUFB6* SNP allele modulates responder status to changes in physical activity. Associations of changes in PA levels and insulin sensitivity **(A, B)** as well as liver fat estimates **(C,D)** are displayed for patients with type 2 diabetes, stratified according to genotype. Type 2 diabetes carriers of the G/G allele of the rs540467 single nucleotide polymorphism of the *NDUFB6* gene are marked as dots (**•**), carriers of the G/A and A/A genotype are marked as crosses (+). The solid regression line depicts a strong trend or a significant association whereas the dotted line represents non-significant associations. Change in insulin sensitivity on the y-axis refer to ln M-value difference while change in physical activity on the x-axis refers to the Baecke index difference.

Based on previous findings showing *NDUFB6* gene polymorphisms modulating responses to exercising in patients with T2D (7, 9), and the observed association of changes in PA and insulin sensitivity in these patients only, we stratified further analyses by the rs540467 SNP. Individuals with T2D carrying the G/G genotype of the *NDUFB6* SNP rs540467 exhibited a positive correlation between changes in M-value and PA levels compared to carriers of the A-allele showing no such association of these variables (**Figures 2A, B**). A linear multivariable model revealed a trend for interaction of the different alleles with PA-mediated changes in insulin sensitivity (p=0.07). Of note, the genotype frequency of the A allele of rs540467 previously associated to non-response to exercise training was 38% in Q2-Q4 and 22% in Q1-Q3 (p=0.12). We further found a decrease of waist circumference (p=0.03, β=-1.52 ± 0.68) and FLI (**Figures 2C, D**) with increasing PA in T2D individuals carrying the G/G allele, but not in A allele carriers. In addition, there were significant interactions between genotypes and PA-mediated changes of waist circumference and FLI (both p<0.05). No difference were found between SNP carriers at baseline or at the follow-up time point regarding the use of medication (**Supplemental Table 1**), dietary composition and energy intake (**Supplemental Table 2**).

### NDUFB6 Modulates Contraction-Induced Changes in Mitochondrial Function and Insulin Signaling in C2C12 Myocytes

**Figure 3:**
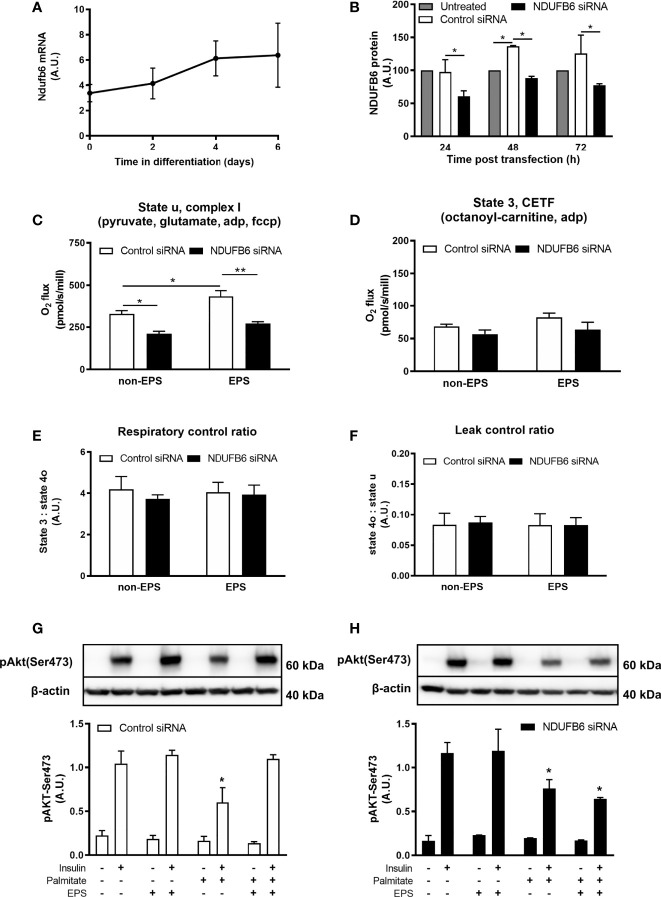
*Ndufb6* expression after siRNA silencing and effects of the *Ndufb6* silencing on mitochondrial function in differentiated C2C12 myotubes after EPS-induced contractions. *Ndufb6* expression during differentiation of C2C12 myocytes **(A)** and protein levels **(B)** after siRNA silencing. *Ndufb6* protein levels were normalized to GAPDH. State u, complex I-linked **(C)** respiration and electron-transferring flavoprotein complex (CETF)-linked **(D)** respiration as well as respiratory control ratio **(E)** and leak control ratio **(F)** were assessed at the basal (non-EPS) and EPS-induced contractions; an independent samples t-test was used for group-comparison. Insulin-stimulated Akt phosphorylation at Ser473 (pAkt-Ser473) after palmitate incubation and electric pulse stimulation (EPS)-induced contractions in differentiated C2C12 myotubes treated with control siRNA **(G)** or *NDUFB6* siRNA **(H)**; Data are expressed as mean ± SEM (n=3-4/group), *p < 0.05 and **p < 0.01 *vs.* corresponding basal and control or untreated condition.

In light of the association of the *NDUFB6* SNP with the M-value, which mainly reflects muscle insulin sensitivity (32), and the previously reported lower mRNA and protein levels of *NDUFB6* in muscles of humans with higher age or T2D (33, 34), we next examined the effects of reduced *NDUFB6* expression in differentiated C2C12 myotubes, a cell culture model suitable to assess effects of PA *in vitro* (12). *Ndufb6* was silenced on day 4 of myoblast differentiation, as the expression of *NDUFB6* mRNA was maximal and remained stable from day 4 on (**Figure 3A**). Treatment for 24 h with siRNA, but not with control RNA reduced *Ndufb6* protein levels by 40% (**Figure 3B**). Thus, all following experiments were performed 24 h after *Ndufb6* silencing.

Compared to control, *Ndufb6* silencing decreased complex I-linked state u respiration by 36% in the absence of EPS-induced contractions (**Figure 3C**). Interestingly, EPS-induced contractions increased complex I-linked respiration in control, but not in *Ndufb6* silenced myotubes (**Figure 3C**). *Ndufb6* silencing neither affected CETF-linked respiration (**Figure 3D**) nor RCR (**Figure 3E**) or LCR (**Figure 3F**), indicators of mitochondrial coupling efficiency and proton leak. Taken together, *in vitro* inhibition of NDUFB6 leads to reduced mitochondrial respiration in this model.

We then tested the effects of EPS on insulin signaling after palmitate-induced insulin resistance. Palmitate inhibited insulin-stimulated pAkt-Ser473 in control myotubes, which was restored to control conditions (without palmitate) after EPS-induced contractions (**Figure 3G**). In contrast, EPS did not rescue palmitate-induced reduction of pAkt-Ser473 in *Ndufb6*-silenced myotubes (**Figure 3H**). Palmitate-induced reduction in pAkt-Ser473 was decreased by 35% in *Ndufb6* siRNA when compared to control siRNA conditions after EPS exposure (0.64 ± 0.02 *vs.* 1.10 ± 0.05 AU; p<0.01), indicating that contractions were not able to protect against palmitate-induced insulin resistance after *Ndufb6* silencing (**Figures 3G, H**).

## Discussion

This study shows that (i) habitual PA is lower in T1D and T2D within the first year after diagnosis, (ii) increases in habitual PA over 5 years do not associate with improvements of insulin sensitivity in 36% of individuals with T2D, (iii) rs540467 SNP of the *NDUFB6* gene is associated with PA-mediated changes in insulin sensitivity, body composition and liver fat estimates (iv) silencing *NDUFB6* in myotubes lowers mitochondrial respiration and inhibits the contraction-mediated rescue from palmitate-induced insulin resistance. These findings suggest that a polymorphism related to mitochondrial function could contribute to modulating the effect of PA on important metabolic endpoints in T2D.

This study extends findings from previous observations of lower habitual PA in individuals with long-standing T1D (35) and T2D (36) to humans with recent-onset diabetes compared to healthy humans when adjusted for age, BMI and sex. Although PA levels are inversely correlated with BMI, the present results are not simply due to differences in FM, but rather result from lower insulin sensitivity in the diabetes groups. Five years after diagnosis, changes in self-reported habitual PA differently associated with improved physical performance in both diabetes groups, but with lower BW, FLI as well as waist circumference and a trend for higher insulin sensitivity only in T2D. Exercise training enhances physical fitness and insulin sensitivity in patients with T2D (1), but even light PA such as supervised walking can improve insulin sensitivity (37). PA can further impact on hepatocellular lipid content (11), which is in agreement with the present findings. We show that the *NDUFB6* SNP exhibits allele-specific modulations of PA regarding this endpoint. This could be driven by alterations in adiposity and visceral fat, as reflected by changes in BW and waist circumference in the G/G allele carriers. Of note, the present study showed that more than one third of patients failed to improve insulin sensitivity despite increased habitual PA. Our non-responder group primarily increased their sports activity, which may confer an insufficient dose, i.e. number, duration or intensity of exercise sessions per week. Indeed, a recent study showed that increasing the dose of exercise can overcome non-response (38). This suggests that a higher dose of PA, as observed in the responders, might also contribute to reduction of non-response.

Despite non-response with regard to insulin sensitivity, improved aerobic fitness suggests that non-response to increased PA is not necessarily a general phenomenon, in line with previous observations (6). In the context of PA and exercise training, the term “non-response” therefore requires a precise definition of an endpoint.

Although medication can interfere with metabolic adaptations to exercise (39), we did not identify differences in medication between SNP carriers and can therefore exclude that medication influences on these associations (**Supplemental Table 1**). Dietary composition and energy intake are other important modulators of glucose metabolism and insulin sensitivity (40, 41), but we also did not find any respective group differences between SNP carriers (**Supplemental Table 2**).

Aside from exogenous factors, inherited factors may contribute to modulating exercise effects on insulin sensitivity, body composition and liver fat content. To our knowledge, this is the first evidence for the *NDUFB6* tag SNP in modulating PA-mediated effects on metabolic endpoints. The results extend our previous data from short-term (9) or long-term training (7) to habitual PA. In these studies, relatives of individuals with T2D carrying the A allele of the *NDUFB6* SNP, rs540467, failed to increase their muscle ATP synthase flux during short-term training (9). Similarly, relatives carrying the G allele of the *NDUFB6* SNP exhibited greater improvement of muscle ATP synthase flux after 26 weeks of exercise training (7), underlining the importance of muscle mitochondrial function. In a previous exercise intervention study, T2D non-responders to 10 weeks of training with regard to muscle mitochondrial function also did not improve their insulin sensitivity (42). We have previously shown that reduced mitochondrial fitness is associated with insulin resistance and T2D (43). However, evidence for an explanation of this dissociation of responses between muscle mitochondrial function and insulin resistance is still scant. Since NDUFB6 is part of the oxidative phosphorylation system with its expression declining under insulin resistant conditions (33, 34), polymorphisms of this gene could modulate the PA-mediated inability to improve insulin sensitivity.

Although the heritability estimates for *NDUFB6* expression have been shown to be up to 65%, both genetic and nongenetic factors likely influence *NDUFB6* expression in skeletal muscle (34). In light of the multifactorial pathogenesis of T2D, this study proposes a possible framework, by which reduced complex I-related respiration relate to insulin sensitivity by silencing *Ndufb6* in myocytes. These findings identify muscle mitochondrial fitness as key components in maintaining whole-body metabolism and underline the significance of an SNP in a gene relevant for energy metabolism.

**Figure 4:**
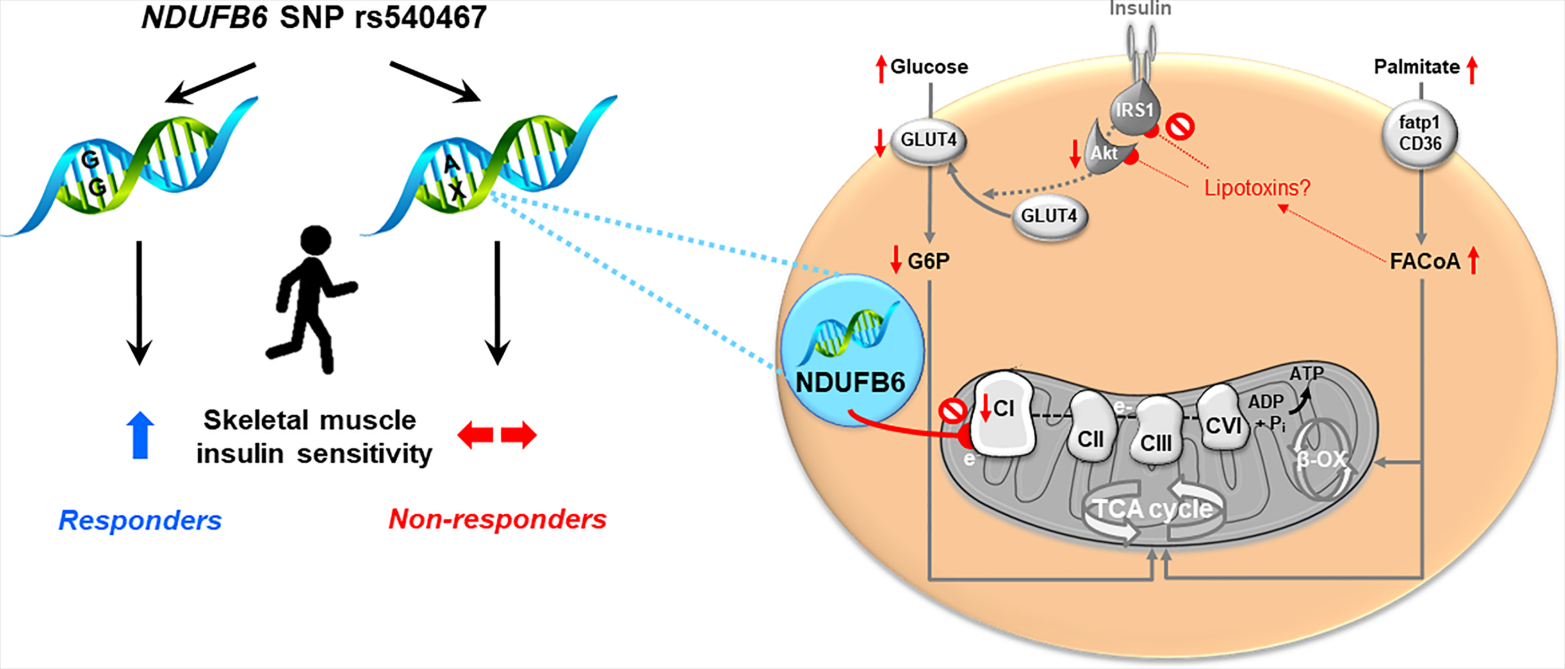
Proposed framework of *NDUFB6* interaction with physical activity-mediated improvement of mitochondrial function.

The study has some limitations. Non-response to PA can be a dose-related phenomenon (38). As PA behavior was assessed by a questionnaire, it is not possible to provide the actual dose of PA in the present cohort. An inherent limitation of the GDS, as an ongoing actively recruiting observational study, is the fact that there are more volunteers included at baseline than at follow up as only a limited number of patients have reached 5 years disease duration at the time of analysis. A further limitation is absence of information on PA and metabolic parameters of those few, who were lost during follow up (~13%). Further to that, we are aware that cell culture is not a perfect surrogate resembling the human situation but we believe that these experiments still provide valuable mechanistic insights. In conclusion, a large percentage of patients with recent-onset T2D does not respond to increased habitual PA with improved insulin sensitivity. The A allele of the *NDUFB6* rs540467 SNP at least partly contributes to this non-response. Silencing of *NDUFB6* affects complex I-mediated mitochondrial respiration, which associates with impaired insulin signaling (**Figure 4**). Thus, our study reveals a relevant gene-environment interaction for one single SNP involved in muscle energy metabolism in T2D, likely affecting muscle mitochondrial function under exercising conditions (44, 45). Sedentary individuals may profit from lifestyle interventions and should be encouraged to increase PA levels early on. Of note, considering the relatively low percentage of non-responders (36%) compared to exercise interventions makes habitual physical activity an attractive alternative to structured exercise. Nevertheless, it becomes increasingly important for clinicians to identify and monitor non-responders to lifestyle interventions and potentially consider specific gene analysis paving the way to precision medicine in the field of T2D.

## Data Availability Statement

The data sets generated during and/or analyzed during the current study are not publicly available, since they are subject to national data protection laws and restrictions imposed by the ethics committee to ensure data privacy of the study participants. However, they can be applied for through an individual project agreement with the principal investigator of the German Diabetes Study. Requests to access the datasets should be directed to michael.roden@ddz.de.

## Ethics Statement

This study was conducted after the approval of the ethics board of Heinrich Heine University Düsseldorf (previous reference number 2478, current reference number 4508) and has been performed in accordance with the ethical standards as set down in the 1964 Declaration of Helsinki and its last amendments of 2013 or comparable ethical standards. All volunteers gave informed consent to the approved protocol. The patients/participants provided their written informed consent to participate in this study.

## THE GDS GROUP

The GDS Group consists of A.E. Buyken, B. Belgardt, G. Geerling, H. Al-Hasani, C. Herder, A. Icks, J. Kotzka, O. Kuss, E. Lammert, JH. Hwang, D. Markgraf, K. Müssig, W. Rathmann, J. Szendrödi, D. Ziegler and M. Roden (speaker).

## Author Contributions

DP, TJ, and MR wrote the manuscript and researched data. PB and KS performed the statistical analyses. O-PZ, SG, KB, YK, NK, NL, DM, VB, KM, JK, BK, JE, and JS researched data, contributed to the discussion and reviewed/edited the manuscript. All authors critically reviewed the manuscript. MR is the guarantor of this work and, as such, had full access to all the data in the study and takes responsibility for the integrity of the data and the accuracy of the data analysis. All authors contributed to the article and approved the submitted version.

## Funding

This work was supported by the Ministry of Culture and Science of the State of North Rhine-Westphalia (MKW NRW) and the German Federal Ministry of Health (BMG). This study was supported in part by a grant of the Federal Ministry for Education and Research (BMBF) to the German Center for Diabetes Research (DZD e.V.) MR is further supported by grants from the European Regional Development Fund (EFRE-0400191), German Research Foundation (DFG, SFB 1116/2). NK received an Albert Renold Travel Fellowship from the European Foundation for the Study of Diabetes (EFSD), TJ a grant from the Deutsche Diabetes Gesellschaft (DDG) Allgemeine Projektförderung and DP from the Dr. Eickelberg-Stiftung. The funding sources had neither influence on design and conduct of this study, collection, analysis and interpretation of the data; nor on the preparation, review, or approval of this article.

## Conflict of Interest

MR receives research support by the Ministry of Culture and Science of the State of North Rhine-Westphalia and the German Federal Ministry of Health, serves as investigator of studies supported by Boehringer-Ingelheim Pharma, Nutriticia/Danone and Sanofi and has served as advisor/consultant for Bristol-Myers Squibb, Eli Lilly, Gilead, Intercept Pharma, Novo Nordisk, Novartis, Poxel, Prosciento, Sanofi, Servier and TARGET NASH.

The remaining authors declare that the research was conducted in the absence of any commercial or financial relationships that could be constructed as a potential conflict of interest.

## Publisher’s Note

All claims expressed in this article are solely those of the authors and do not necessarily represent those of their affiliated organizations, or those of the publisher, the editors and the reviewers. Any product that may be evaluated in this article, or claim that may be made by its manufacturer, is not guaranteed or endorsed by the publisher.

## Supplementary Material

Supplementary Table 1 AB: Differences in medication between G/G allele as well as A/G and A/A allele carriers at baseline **(A)** and at the follow-up time point **(B)**. NSAID, Nonsteroidal anti-inflammatory drugs.

Supplementary Table 2 Intake of dietary macronutrients of a subset of individuals with type 2 diabetes (n=37) at **(A)** baseline and after 5 years follow-up and between different genotype carriers at **(B)** baseline and **(C)** after 5 years follow-up. Data are given as mean ± standard deviations (SD); p-values have been assessed with dependent samples t-test.

## Abbreviations

ALT, Alanine aminotransferase; BIA, Bioimpedance analysis; BW, body weight; EPS, electronic pulse stimulation; FCCP, carbonyl-cyanide-p-trifluoromethoxyphenylhydrazone; FLI, fatty liver index; FM, fat mass; GDS, German Diabetes Study; hsCRP, high-sensitivity C-reactive protein; LCR, leak control ratio; NDUFB6, NADH dehydrogenase 1 beta subcomplex subunit 6; PA, physical activity; RCR, respiratory control ratio; SNP, single nucleotide polymorphism; VO_2_AT, VO_2_ at the aerobic threshold.
