# Supplementary material for: NDUFB6 Polymorphism Is Associated With Physical Activity-Mediated Metabolic Changes in Type 2 Diabetes
Source: Front Endocrinol (Lausanne). 2021 Sep 24;12:693683. doi: 10.3389/fendo.2021.693683 (PMC8518618; doi:10.3389/fendo.2021.693683)
Supplement: Supplementary Table 1 — AB: Differences in medication between G/G allele as well as A/G and A/A allele carriers at baseline (A) and at the follow-up time point (B). NSAID, Nonsteroidal anti-inflammatory drugs. [file Table_1.docx]

**Supplemental Table 1AB: Differences in medication between G/G allele as well as G/A and A/A allele carriers at baseline (A) and at the follow-up time point (B).**

A

| **Medication baseline** | **G/G allele carriers (%)** | **G/A and A/A allele carriers (%)** | **p-value** |
| --- | --- | --- | --- |
| Insulin | 11 | 4 | 0.43 |
| Metformin | 56 | 59 | 0.82 |
| Sulfonylureas | 8 | 4 | 0.66 |
| GLP-1 receptor agonists | 0 | 0 | 1.00 |
| DPP4 inhibitors | 2 | 4 | 0.51 |
| SGLT2 Inhibitor | 0 | 0 | 0.81 |
| Statins | 16 | 11 | 0.74 |
| Beta-blocker | 19 | 19 | 1.00 |
| NSAID | 5 | 7 | 0.63 |

B

| **Medication follow-up** | **G/G allele carriers (%)** | **A/G and A/A allele carriers (%)** | **p-value** |
| --- | --- | --- | --- |
| Insulin | 21 | 7 | 0.21 |
| Metformin | 56 | 67 | 0.36 |
| Sulfonylureas | 8 | 7 | 0.98 |
| GLP-1 receptor agonists | 0 | 0 | 1.00 |
| DPP4 inhibitors | 6 | 4 | 0.97 |
| SGLT2 Inhibitor | 0 | 0 | 1.00 |
| Statins | 16 | 19 | 0.76 |
| Beta-blocker | 24 | 22 | 0.98 |
| NSAID | 3 | 7 | 0.58 |
