# Supplementary material for: NDUFB6 Polymorphism Is Associated With Physical Activity-Mediated Metabolic Changes in Type 2 Diabetes
Source: Front Endocrinol (Lausanne). 2021 Sep 24;12:693683. doi: 10.3389/fendo.2021.693683 (PMC8518618; doi:10.3389/fendo.2021.693683)
Supplement: Supplementary Table 2 — Intake of dietary macronutrients of a subset of individuals with type 2 diabetes (n=37) at (A) baseline and after 5 years follow-up and between different genotype carriers at (B) baseline and (C) after 5 years follow-up. Data are given as mean ± standard deviations (SD); p-values have been assessed with dependent samples t-test. [file Table_2.docx]

**Supplemental Table 2. Intake of dietary macronutrients of a subset of individuals with type 2 diabetes (n=37) at (A) baseline and after 5 years follow-up and between different genotype carriers at (B) baseline and (C) after 5 years follow-up**

**A**

| **Macronutrient** | **Baseline** | **Follow-up** | **p-value** |
| --- | --- | --- | --- |
| Protein (g/day) | 97±31 | 94±30^†^ | 0.40 |
| Fat (g/day) | 115±38 | 117±40 | 0.52 |
| Carbohydrates (g/day) | 222±97 | 227±87 | 0.16 |
| Total energy (kcal/day) | 2406±824 | 2445±755 | 0.35 |

**B**

| **Macronutrient** | **G/G allele carriers** | **G/A and A/A allele carriers** | **p-value** |
| --- | --- | --- | --- |
| Protein (g/day) | 89±29 | 98±27 | 0.37 |
| Fat (g/day) | 106±34 | 125±38 | 0.17 |
| Carbohydrates (g/day) | 196±77 | 224±66 | 0.30 |
| Total energy (kcal/day) | 2170±723 | 2539±656 | 0.16 |

**C**

| **Macronutrient** | **G/G allele carriers** | **G/A and A/A allele carriers** | **p-value** |
| --- | --- | --- | --- |
| Protein (g/day) | 97±32 | 87±25 | 0.10 |
| Fat (g/day) | 119±43 | 115±36 | 0.64 |
| Carbohydrates (g/day) | 232±96 | 211±59 | 0.22 |
| Total energy (kcal/day) | 2484±818 | 2338±609 | 0.35 |

Data are given as mean ± standard deviations (SD). Significant differences as determined by dependent samples t-test;
